# Electric-Field-Controlled Rotational Spectroscopy for Highly Selective Gas Sensing

**DOI:** 10.3390/s26206363

**Published:** 2026-10-09

**Authors:** Zorica Branković, Goran Branković, Željko Despotović, Yuri Rostovtsev

**Affiliations:** 1 University of Belgrade - Institute for Multidisciplinary Research, National Institute of the Republic of Serbia, Kneza Višeslava 1, 11030 Belgrade, Serbia; zorica.brankovic@imsi.bg.ac.rs (Z.B.); goran.brankovic@imsi.bg.ac.rs (G.B.); 2 University of Belgrade - Institute Mihailo Pupin, Volgina 15, 11000 Belgrade, Serbia; zeljko.despotovic@pupin.rs; 3 Department of Physics and Center for Nonlinear Sciences, University of North Texas, 1155 Union Cir, Denton, TX 76203, USA

**Keywords:** molecular gas detection, molecular rotational levels, Stark effect

## Abstract

Air quality control is essential for preventing human exposure to toxic and harmful gases and therefore requires reliable and highly selective gas sensors. Over the past few decades, numerous sensing techniques based on different physical and chemical interactions with sensitive materials have been developed. These include optical, calorimetric, acoustic, and electrical sensing methods such as metal-oxide semiconductor sensors. Although significant progress has been achieved in sensitivity—reaching detection limits at the parts-per-billion level—the precise analysis of complex gas mixtures remains a major challenge due to limited selectivity and cross-sensitivity. In this work, we propose a new gas-sensing mechanism based on the interaction of molecular rotational states with a quasistatically swept DC electric field. For molecules possessing permanent electric dipole moments, the presence of a static electric field produces Stark splitting of rotational levels that is characteristic of each molecular species. The proposed scheme enables a single-frequency detector to analyze gas mixtures with high selectivity, accuracy, and sensitivity. The sensing mechanism relies on species-specific Stark interactions and resonant coupling of molecular dipoles to the applied electric field. Preliminary calculations are presented for the molecules H_2_O and NH_3_, illustrating the feasibility of the method. These results suggest that electric-field-controlled rotational spectroscopy provides a promising pathway toward highly selective gas sensing in complex molecular environments.

## 1. Introduction

The composition of the Earth’s atmosphere has evolved continuously over geological timescales as a result of natural processes and biological activity. Variations in oxygen concentration and the emergence of new organisms gradually established environmental conditions suitable for the development of life as we know it today. Although such global transformations occur over long timescales, significant changes in atmospheric composition can also arise locally and rapidly due to pollution and the release of toxic gases. The presence of hazardous gases poses serious risks to human health, industrial safety, and environmental stability. Consequently, reliable air-quality monitoring and early detection of harmful gases are essential for preventing environmental damage and maintaining the balance of natural ecosystems.

Gas sensing technologies aim to convert gas-induced physical or chemical changes into measurable signals. Over the past several decades, a wide range of sensing mechanisms have been developed for molecular detection [1,2,3,4,5,6]. These include optical techniques [7,8], calorimetric sensors [9], acoustic spectroscopy [10], and methods based on variations in electrical properties such as metal-oxide semiconductor sensors [11]. Continuous progress in sensor design has led to remarkable improvements in sensitivity, in some cases reaching the parts-per-billion (ppb) level [12,13]. Despite these advances, however, two fundamental challenges remain: limited selectivity and cross-sensitivity in complex gas mixtures. When multiple molecular species with similar chemical or spectroscopic properties are present, reliable identification becomes increasingly difficult.

Developing highly selective sensors capable of analyzing multicomponent gas mixtures therefore remains a major challenge in gas detection technology. Such sensors would have broad applications ranging from environmental monitoring and industrial process control to biomedical diagnostics and atmospheric science. As a result, there is a continuing search for new sensing mechanisms that can overcome the intrinsic limitations of existing approaches. In particular, it is desirable to develop techniques that allow selective detection of specific molecular species while simultaneously enhancing the detection signal toward the theoretical limits of molecular absorption.

Spectroscopic gas sensing relies on the fact that each molecule possesses a unique set of spectral transitions. Optical radiation excites electronic transitions, while infrared and Raman spectroscopy exploit the vibrational structure of molecules [14,15]. Rotational transitions, in contrast, occur in the far-infrared or microwave spectral range. For electronic and vibrational transitions, the thermal conditions $kT\ll \hbar \omega_{\mathrm{opt}}$ and $kT∼\hbar \omega_{\mathrm{vib}}$ often lead to large population differences between molecular levels, resulting in strong absorption signals. In contrast, for rotational transitions at ambient temperature, the condition $kT\gg \hbar \omega_{\mathrm{rot}}$ typically holds, leading to very small population differences and consequently weak absorption. This significantly limits the sensitivity of conventional rotational spectroscopy for gas detection.

In this work, we propose a fundamentally different gas-sensing mechanism based on the interaction of a quasistatically swept electric field with the rotational structure of polar molecules [6,16,17]. Molecules possessing permanent electric dipole moments exhibit rotational spectra that are highly species-specific. By applying a quasistatically swept DC electric field, the rotational energy levels can be shifted through the Stark interaction, thereby modifying the transition frequencies and population differences between the rotational states.

An important mechanism enabling control of molecular rotational populations is the Stark effect [18] produced by an external electric field. For polar molecules with a permanent electric dipole moment, interaction with a DC electric field leads to shifts and mixing of rotational energy levels. In the rigid-rotor approximation, the molecular Hamiltonian can be written as

(1) $$\hat{H}=A{\hat{J}}_{a}^{2}+B{\hat{J}}_{b}^{2}+C{\hat{J}}_{c}^{2}-℘\cdot E,$$

where *A*, *B*, and *C* are the rotational constants, *℘* is the molecular dipole moment, and E is the applied electric field. For symmetric molecules such as NH_3_, A=B, whereas for asymmetric molecules such as H_2_O, the three rotational constants are distinct (A≠B≠C). The Stark interaction couples rotational states with ΔJ=±1, thus modifying the population distribution among rotational levels and inducing partial alignment of molecular dipoles along the field. When the DC electric field is varied sufficiently slowly, the rotational populations are assumed to follow the stationary Boltzmann distribution corresponding to the instantaneous field-dependent energy levels. The proposed mechanism enables highly selective gas sensing by using a single low-frequency electric-field excitation.

The remainder of this paper is organized as follows. Section 2 presents the theoretical model describing the interaction of polar molecules with an external electric field and develops the proposed bridge-based detection scheme. Section 3 discusses the sensing mechanism, its potential applications, practical limitations, and possible experimental implementations.

## 2. Materials and Methods

### 2.1. Physical Principles of Electric-Field-Driven Rotational Sensing

The molecular Hamiltonian shown in Equation (1) can be rewritten as

(2) $$\hat{H}=\frac{A+B}{2}{\hat{J}}^{2}+\left(C-\frac{A+B}{2}\right){\hat{J}}_{c}^{2}+\frac{A-B}{4}\left({\hat{J}}_{-}^{2}+{\hat{J}}_{+}^{2}\right)-℘\cdot E$$

where $\hat{\vec{J}}$ is the operator of the total molecular angular momentum, ${\hat{J}}_{\pm }={\hat{J}}_{a}\pm i{\hat{J}}_{b}$, and ${\hat{J}}_{c}$ is the projection of the angular momentum onto the molecular *c* axis in the molecular frame. The states |JKM〉 are characterized by the quantum numbers *J*, *K*, and *M*, defined by

$${\hat{J}}^{2}|JKM\rangle =\hbar^{2}J\left(J+1\right)|JKM\rangle ,\;{\hat{J}}_{z}^{\prime }|JKM\rangle =\hbar M|JKM\rangle ,\;{\hat{J}}_{c}|JKM\rangle =\hbar K|JKM\rangle ,$$

where ${\hat{J}}_{z}^{\prime }$ is the projection of the angular momentum onto the *z* axis in the laboratory frame and ${\hat{J}}_{c}$ is its projection onto the molecular *c* axis (see [19] for more details).

A symmetric-top molecule is NH_3_, in which the three hydrogen atoms form an equilateral triangle and the nitrogen atom is located above the plane defined by the hydrogen atoms. Let the N–H bond length be *r* and the H–N–H bond angle be γ. For ammonia, r≃1.012Å, $\gamma ≃106.7^{\circ }$.

The moment of inertia about the symmetry axis, $I_{\|}=4.46\times 10^{-47}\;\;\mathrm{kg}\;m^{2}$, is determined solely by the three hydrogen atoms, since the nitrogen atom lies on the symmetry axis and therefore does not contribute to this moment of inertia. The corresponding rotational constant is $C=\frac{h}{8\pi^{2}I_{c}}≃187\;\mathrm{GHz}$. For an axis perpendicular to the symmetry axis, $I_{a}=I_{b}$. The perpendicular moment has two contributions: the transverse distribution of the three hydrogen atoms and the displacements of the nitrogen atom and the H_3_ plane relative to the center of mass, and $A=B=\frac{h}{8\pi^{2}I_{a}}≃298\;\mathrm{GHz}$ (see Table 1).

The permanent electric dipole moments and rotational constants *A*, *B*, and *C* used in the calculations are taken from established spectroscopic data sources and experimental literature, as indicated by the corresponding references in Table 1. These parameters determine the field-free rotational energy structure and the magnitude of the Stark interaction and therefore provide the molecular-specific input parameters for the calculations presented below.

The rotational energy of an oblate symmetric-top molecule is then

(3) $$E_{J,K,M}=hBJ\left(J+1\right)+h\left(C-B\right)K^{2},$$

where *K* is the projection of J onto the molecular symmetry axis.

The interaction of a molecule with an external static electric field is described by the Stark Hamiltonian

(4) $$H_{\mathrm{Stark}}=-℘\cdot E.$$

The matrix elements of the total Hamiltonian (see Equation (2)), including the Stark interaction, were evaluated in the |JKM〉 basis. The resulting Hamiltonian matrix was then diagonalized to obtain the Stark-shifted energy eigenvalues $E_{i}$ and the corresponding eigenstates. The new eigenstates are expressed as linear combinations of the field-free rotational basis states,

(5) $$|i\rangle =\sum_{JKM}c_{i}^{JKM}\;|JKM\rangle ,$$

where $c_{i}^{JKM}$ are the expansion coefficients determined by the diagonalization procedure.

The permanent electric dipole of the ammonia molecule is ${℘}_{NH_{3}}=1.47$ D, and the interaction with a static electric field leads to the splitting of the rotational molecular levels as

$$\Delta \nu_{100}=\frac{E_{100}-E_{10\pm 1}}{h}=0.75{\left(E_{0}\left[\mathrm{kV}/\mathrm{cm}\right]\right)}^{2}\;MHz,$$

$$\Delta \nu_{110}=\frac{E_{110}-E_{11\pm 1}}{h}=0.37\cdot E_{0}\left[V/\mathrm{cm}\right]\;MHz.$$

**Figure 1:**
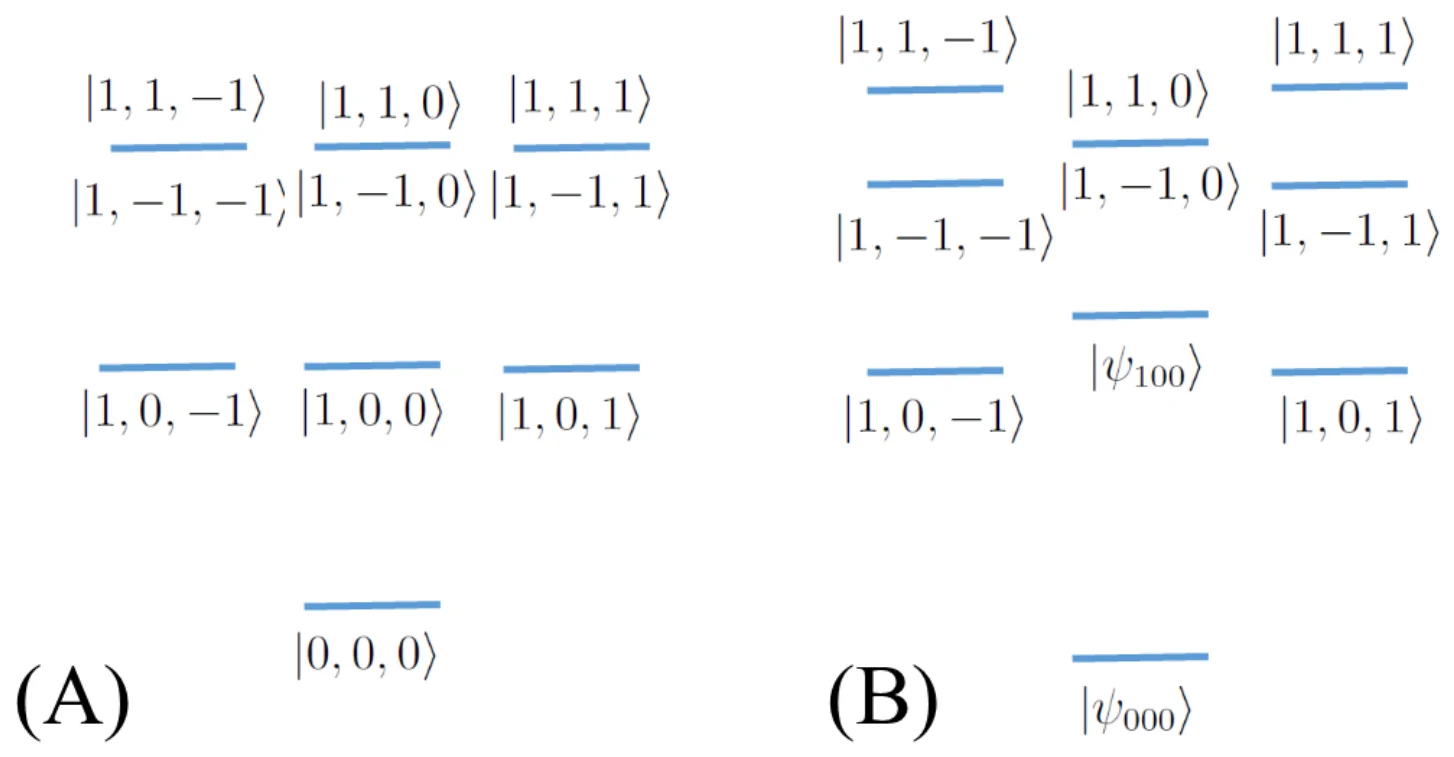
Rotational energy-level structure of the symmetric-top NH_3_ molecule: (**A**) field-free rotational levels; (**B**) Stark splitting of the rotational levels in the presence of an external DC electric field.

As shown in Figure 1, panel (A) presents the rotational level structure of $NH_{3}$ without the DC electric field, whereas panel (B) illustrates the splitting of the levels due to the DC electric field (see Table 2).

Magnetic molecular moments are given by

(6) $${\hat{\mu }}_{j}=\frac{1}{2}\sum_{i}q_{i}{\vec{r}}_{i}\times \left(\frac{{\vec{J}}_{j}}{I_{j}}\times {\vec{r}}_{i}\right)$$

and the components of the magnetic moment for $NH_{3}$ are given by ${\hat{\mu }}_{a}=0.96\frac{q}{2M_{p}c}{\hat{J}}_{a}$, ${\hat{\mu }}_{b}=0.96\frac{q}{2M_{p}c}{\hat{J}}_{b}$, ${\hat{\mu }}_{c}=\frac{q}{2M_{p}c}{\hat{J}}_{c}$, where $M_{p}$ is the proton mass. Finally, the total magnetic moment of an NH_3_ molecule can be approximated as

(7) $$\hat{\mu }≃\frac{q}{2M_{p}c}\hat{J},$$

which determines the magnetic response of the NH_3_ molecule.

H_2_O is an asymmetric-top molecule. Its rotational constants are $A=\frac{h}{8\pi^{2}I_{a}}$, $B=\frac{h}{8\pi^{2}I_{b}}$, and $C=\frac{h}{8\pi^{2}I_{c}}$, where $I_{a}<I_{b}<I_{c}$ are the principal moments of inertia. Because the molecule is planar, one principal axis is perpendicular to the molecular plane. With the molecule in the xz-plane, this is the *y*-axis. The moment about the *y*-axis is $I_{y}=I_{x}+I_{z}$. Thus, $I_{a}=I_{x}$, $I_{b}=I_{z}$, $I_{c}=I_{y}$, with $I_{a}<I_{b}<I_{c}$. One finds $I_{a}≃1.02\times 10^{-47}\;\mathrm{kg}\;m^{2}$, $I_{b}≃1.92\times 10^{-47}\;\mathrm{kg}\;m^{2}$, and $I_{c}≃2.94\times 10^{-47}\;\mathrm{kg}\;m^{2}$. Therefore, $A=\frac{h}{8\pi^{2}I_{a}}≃836\;\mathrm{GHz}$, $B=\frac{h}{8\pi^{2}I_{b}}≃435\;\mathrm{GHz}$, $C=\frac{h}{8\pi^{2}I_{c}}≃278\;\mathrm{GHz}$ (see Table 1).

Water is an asymmetric-top molecule whose rotational Hamiltonian is given by Equation (1). For A≠B≠C, the states |JKM〉 can be used as basis states for constructing the molecular Hamiltonian. Diagonalization of the resulting Hamiltonian matrix yields the Stark-shifted energy levels and the corresponding eigenstates, as expressed in Equation (5).

The permanent dipole moment of water is ${℘}_{H_{2}O}=1.85\;D$. The interaction with a static electric field leads to the splitting of the rotational molecular levels as

$$\Delta \nu_{100}=\frac{E_{100}-E_{10\pm 1}}{h}=0.59{\left(E_{0}\left[\mathrm{kV}/\mathrm{cm}\right]\right)}^{2}\;MHz,$$

$$\Delta \nu_{110}=\frac{E_{110}-E_{11\pm 1}}{h}=1.92{\left(E_{0}\left[\mathrm{kV}/\mathrm{cm}\right]\right)}^{2}\;MHz.$$

**Figure 2:**
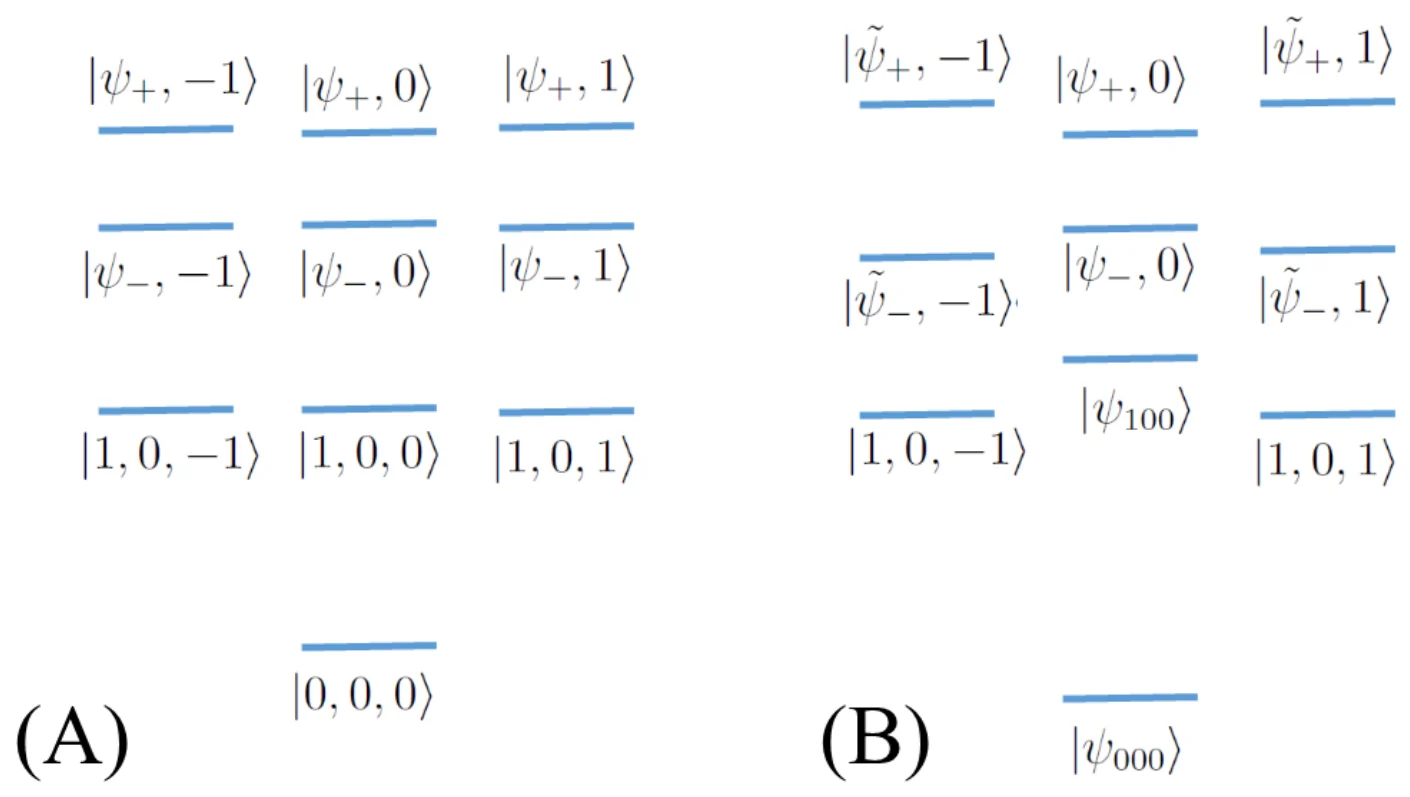
Rotational energy-level structure of the asymmetric-top H_2_O molecule: (**A**) field-free rotational levels; (**B**) Stark splitting of the rotational levels in the presence of an external DC electric field.

As shown in Figure 2, panel (A) presents the rotational level structure of H_2_O in the absence of the DC electric field, whereas panel (B) illustrates the Stark splitting produced by the applied DC electric field (see Table 2).

Using Equation (6), we obtain the components of the magnetic moment for the H_2_O molecule as ${\hat{\mu }}_{a}=0.47\frac{q}{2M_{p}c}{\hat{J}}_{a}$, ${\hat{\mu }}_{b}=0.43\frac{q}{2M_{p}c}{\hat{J}}_{b}$, ${\hat{\mu }}_{c}=\frac{q}{2M_{p}c}{\hat{J}}_{c}$. The total magnetic moment can therefore be approximated as

(8) $$\hat{\vec{\mu }}≃\frac{q}{4M_{p}c}\left(\hat{\vec{J}}+{\hat{\vec{J}}}_{c}\right),$$

which determines the magnetic response of the H_2_O molecule.

It is instructive to estimate the magnitude of the DC electric field required to produce appreciable Stark splitting of the rotational levels using the results summarized in Table 2.

For the transition |100〉→|10±1〉, the Stark splitting depends quadratically on the applied DC electric field for both NH_3_ and H_2_O. A splitting of approximately 1 MHz requires electric fields of about 1.15 kV/cm for NH_3_ and 1.3 kV/cm for H_2_O. For a splitting of 1 GHz, the required fields increase to approximately 36.5 kV/cm and 41.1 kV/cm, respectively.

For the transition |110〉→|11±1〉, the Stark splitting is linear in the applied DC electric field for NH_3_, whereas it is quadratic for H_2_O. A splitting of approximately 1 MHz requires a field of only about 2.5 V/cm for NH_3_, compared with approximately 0.7 kV/cm for H_2_O. For a splitting of 1 GHz, the corresponding fields are approximately 2.5 kV/cm and 22.8 kV/cm, respectively.

These estimates illustrate the substantial difference between the linear and quadratic Stark regimes. In particular, a linear Stark effect can produce a given frequency splitting at considerably lower electric fields than a quadratic Stark effect. This feature is especially advantageous for practical sensing applications, since it can significantly reduce the DC electric-field strength required to tune a rotational transition into resonance.

### 2.2. Sensor Signal Formation and Bridge-Based Impedance Detection Scheme

**Figure 3:**
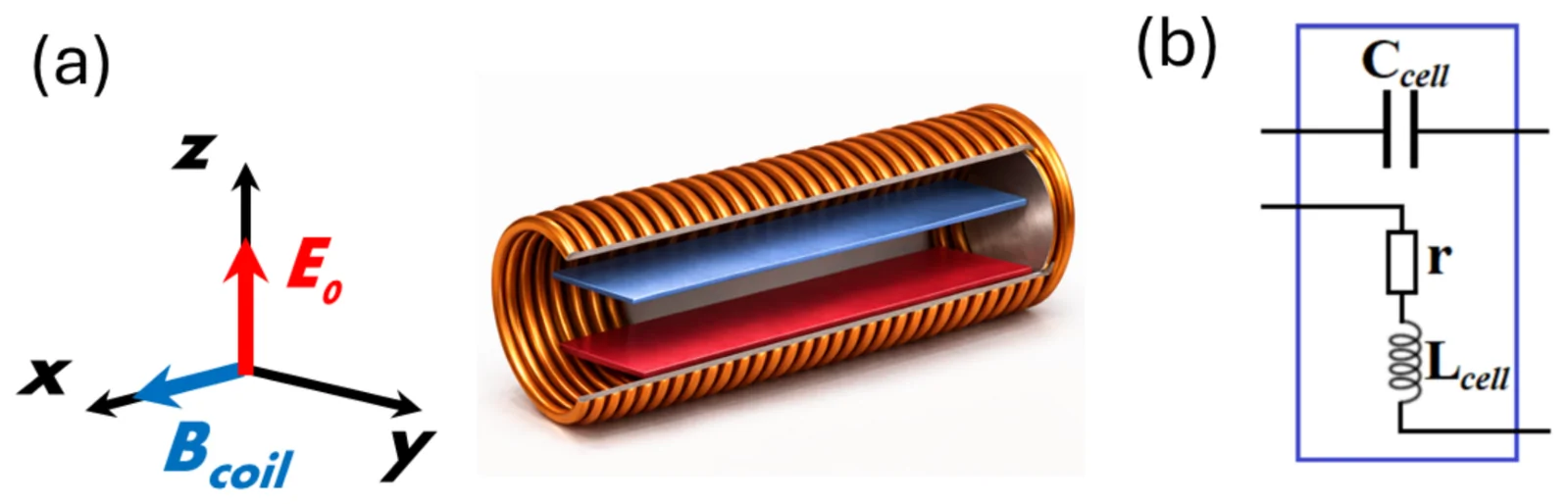
Schematic configuration of the molecular gas sensing cell. (**a**) A solenoid coil surrounding a gas cell containing two parallel-plate electrodes. The electrodes generate a strong DC electric field $E_{0}$ along the *z* axis, while the coil generates an AC magnetic field $B_{\mathrm{coil}}\left(t\right)$ along the *x* axis. Thus, $E_{0}⊥B_{\mathrm{coil}}\left(t\right)$. (**b**) Relative orientation of the applied fields and the corresponding molecular transitions. With the DC electric field defining the quantization axis, the transverse AC magnetic field drives transitions with the selection rule ΔM=±1. This configuration allows the DC electric field to control the Stark splitting of the molecular rotational levels, while the AC magnetic field drives transitions between the Stark-split states.

A gas detection cell equipped with parallel-plate electrodes is used to apply a strong DC electric field $E_{0}$ (see Figure 3). The population of the rotational state |JKM〉 is given by the Boltzmann distribution,

$$N_{JKM}=\frac{N\exp\;\left(-\frac{E_{JKM}}{k_{B}T}\right)}{\sum_{JKM}\exp\;\left(-\frac{E_{JKM}}{k_{B}T}\right)},$$

where *N* is the total number of molecules.

When the DC electric field is applied, the molecular rotational levels are shifted by the Stark effect, as shown in Figure 1 and Figure 2. As a consequence, the thermal populations of the rotational states are redistributed. The population difference between the states

$$|i\rangle =\sum_{JKM}c_{i}^{JKM}\;|JKM\rangle \;and\;|j\rangle =\sum_{JKM}c_{j}^{JKM}\;|JKM\rangle$$

is therefore

$$N_{i}-N_{j}=\frac{N}{\sum_{i}\exp\;\left(-\frac{E_{i}}{k_{B}T}\right)}\left[\exp\;\left(-\frac{E_{i}}{k_{B}T}\right)-\exp\;\left(-\frac{E_{j}}{k_{B}T}\right)\right].$$

For levels satisfying $|E_{i}-E_{j}|\ll k_{B}T$, this expression can be approximated as

$$N_{i}-N_{j}≃-\frac{N}{S_{R}}\exp\;\left(-\frac{E_{i}}{k_{B}T}\right)\frac{E_{i}-E_{j}}{k_{B}T},$$

where

(9) $$S_{R}=\sum_{i}\exp\;\left(-\frac{E_{i}}{k_{B}T}\right)$$

is the rotational partition function. To detect these population differences, a magnetic coil is placed inside the cell, as illustrated in Figure 3. To describe the interaction of the molecules with the magnetic field generated by the coil, we write the total Hamiltonian as

(10) $$\hat{H}={\hat{H}}_{\mathrm{mol}}+{\hat{H}}_{\mathrm{Stark}}+{\hat{H}}_{\mathrm{coil}},$$

where the first two terms describe the molecular rotational structure and its interaction with the static electric field, respectively. The interaction with the magnetic field generated by the coil (see Figure 3) is given by

$${\hat{H}}_{coil}=-\sum_{ij}{\vec{B}}_{coil}\cdot {\vec{\mu }}_{ij}\left|i\rangle \langle j\right|$$

where ${\vec{\mu }}_{ij}$ are the matrix elements of the magnetic moment operators given by Equations (7) and (8). The equation for the density matrix element corresponding to magnetic polarization [24] is

$${\dot{\rho }}_{ij}=-\left(\Gamma +i\left(\omega_{ij}-\omega_{c}\right)\right)\rho_{ij}+i\frac{\mu_{ij}B_{coil}}{\hbar }\left(\rho_{ii}-\rho_{jj}\right)$$

where $\omega_{ij}$ is the transition frequency between states |i〉 and |j〉, $\omega_{c}$ is the frequency of the magnetic field generated by the coil, and Γ is the coherence relaxation rate.

In the weak-field limit, $\mu_{ij}B_{\mathrm{coil}}\ll \hbar \Gamma$, the steady-state solution is given by

(11) $$\rho_{ij}=\frac{i\mu_{ij}B_{coil}}{\hbar (\Gamma +i\Delta )}\left(\rho_{ii}-\rho_{jj}\right)$$

where $\Delta =\omega_{ij}-\omega_{c}$ is the detuning between the molecular transition frequency and the frequency of the magnetic field generated by the coil. Then, the magnetization of the molecular gas $\vec{M}$ is given by

$$\vec{M}=\sum_{i,j}{\vec{\mu }}_{ij}N\rho_{ij}$$

and

(12) $$\vec{B}=\mu_{0}\left(\vec{H}+\vec{M}\right)=\mu_{0}\left(1+\frac{i\mu_{0}\mu_{ij}^{2}\Delta N}{\hbar (\Gamma +i\Delta )}\right)\vec{H}$$

where $\Delta N=N\rho_{ii}-N\rho_{jj}=N_{i}-N_{j}$ is the population difference between states *i* and *j*. The relative magnetic permeability of molecular gas $\mu_{r}$ is

$$\mu_{r}=1+\frac{i\mu_{0}\mu_{ij}^{2}\Delta N}{\hbar (\Gamma +i\Delta )}$$

Thus, the magnetic susceptibility is given by

$$\chi_{m}=\mu_{r}-1=\frac{i\mu_{0}\mu_{ij}^{2}\Delta N}{\hbar (\Gamma +i\Delta )}$$

The Stark-induced energy shifts also modify the frequencies of the magnetic-dipole rotational transitions (see Figure 1 and Figure 2). When the bridge excitation frequency becomes resonant with one of these transitions, the magnetic susceptibility of the gas changes, resulting in a small variation of the inductance of the sensing coil, as

$$L_{cell}\left(E\right)=L_{cell}^{\left(0\right)}\left(1+\chi_{m}\left(E\right)\right),$$

where $L_{cell}^{\left(0\right)}$ is the inductance of the sensing coil in the absence of the testing gas.

**Figure 4:**
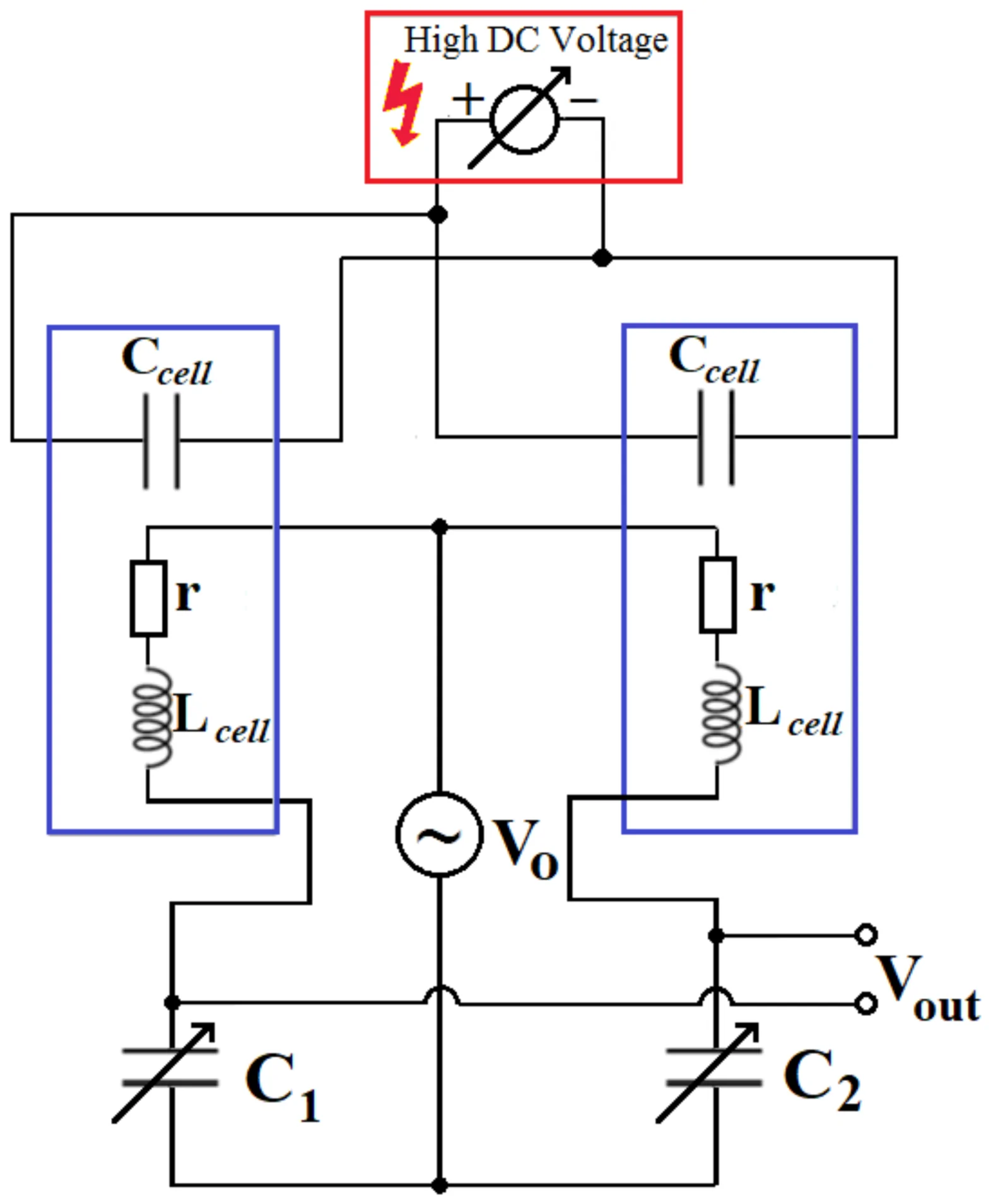
Schematic diagram of the high-voltage bias circuit and the low-voltage LC bridge circuit. The high-voltage DC supply applies a voltage across the capacitors $C_{\mathrm{cell}}$ of cells #1 and #2, generating a DC electric field in each gas cell and producing Stark splitting of the molecular rotational energy levels. The low-voltage AC circuit applies the voltage $V_{0}$ to an LC bridge consisting of capacitors $C_{1}$ and $C_{2}$ and the two cell coils $L_{\mathrm{cell}}$. The coils generate AC magnetic fields that drive rotational transitions of the molecules in the gas cells. By varying the DC electric field, the Stark-shifted molecular transition is tuned into resonance with the frequency of the applied AC field. When one arm of the bridge contains the target gas, the resonant molecular response produces an imbalance of the bridge and a corresponding output voltage $V_{\mathrm{out}}$, providing the signal for selective gas detection.

To detect this small inductance variation with high sensitivity, the bridge circuit shown in Figure 4 is employed. The bridge consists of two identical arms containing matched electrical components, so that in the absence of a molecular resonance the bridge is perfectly balanced and the differential output voltage vanishes,

$$V_{\mathrm{out}}=0.$$

The gas sensing cell (cell #1) is incorporated into one arm of the bridge, while an identical reference cell (cell #2) is placed in the corresponding arm. Two identical capacitors $C_{1}=C_{2}$ complete the symmetric bridge configuration, as shown in Figure 4. In the absence of the applied DC electric field, both coils have identical inductances, and the bridge remains balanced.

When one arm of the bridge contains the target gas, applying the DC electric field shifts the molecular rotational levels through the Stark effect. At a particular value of the electric field, the frequency of a Stark-shifted rotational transition coincides with the operating frequency of the bridge. Under this resonance condition, the inductance of the sensing coil changes by the factor $\left(1+\chi_{m}\right)$, breaking the bridge symmetry and producing a finite output voltage,

$$\Delta V_{\mathrm{out}}\propto \chi_{m}.$$

For a gas mixture, each molecular species is characterized by its rotational constants and permanent electric dipole moment. Consequently, different species generally exhibit different electric-field-dependent resonance conditions. By sweeping the DC electric field while monitoring the bridge output, the resulting resonance features can be used to distinguish molecular species. The degree of selectivity depends on the separation of the resonances, their linewidths, and the composition of the gas mixture. This approach enables simultaneous and selective detection of multiple gases using a single sensing element.

The voltage across the bridge, $V_{out}$, shown in Figure 4, is given by

(13) $$V_{out}=V_{0}\left[\frac{Z_{C_{1}}}{r_{1}+Z_{L_{1}}+Z_{C_{1}}}-\frac{Z_{C_{2}}}{r_{2}+Z_{L_{2}}+Z_{C_{2}}}\right]$$

where $r_{cell\#1}=r_{1}$, $L_{cell\#1}=L_{1}$, $r_{cell\#2}=r_{2}$, $L_{cell\#2}=L_{2}$, $Z_{C_{1,2}}=\frac{1}{i\omega C_{1,2}}$, and $Z_{L_{1,2}}=i\omega L_{1,2}$.

The quality of the resonance can be characterized by the factor $Q\equiv \sqrt{Z_{L}Z_{C}}/r$. Then, for $r_{1}≃r_{2}=r$, $Z_{C1}≃Z_{C2}=Z_{C}$, and $Z_{L1}≃Z_{L2}=Z_{L}$, the voltage $V_{\mathrm{out}}$ across the bridge (see Figure 4) is given by

(14) $$V_{\mathrm{out}}=\underset{Bridge\;imbalance}{\underset{︸}{V_{0}Q\left(\frac{\Delta Z_{C}}{Z_{C}}-\frac{\Delta Z_{C}+\Delta Z_{L}+\Delta r}{r}\right)}}-\underset{molecular\;response}{\underset{︸}{\;\;\;\;\;V_{0}Q^{2}\chi_{m}\;\;\;\;\;}}$$

where $\Delta Z_{C}=Z_{C_{1}}-Z_{C_{2}}$, $\Delta Z_{L}=Z_{L_{1}}-Z_{L_{2}}$, $\Delta r=r_{1}-r_{2}$. The detected voltage consists of the signal contribution and the thermal noise generated by the bridge components:

(15) $$V_{\mathrm{detected}}=V_{0}Q^{2}\frac{\mu_{0}\mu_{ij}^{2}\Delta N}{\hbar \Gamma }\pm V_{T},$$

where $V_{T}$ represents the equivalent thermal noise voltage of the bridge.

The minimum detectable density determined by the Johnson–Nyquist noise ($V_{T}=\sqrt{k_{B}Tr\Delta f}$) is given by

(16) $$N_{min}=\frac{\hbar \Gamma }{\mu_{0}\mu_{ij}^{2}}\frac{kT}{\hbar \omega }\sqrt{\frac{4kT\Delta f}{V_{0}^{2}/r}}{\left(\frac{r}{\omega L}\right)}^{2}S_{R}$$

where $\mu_{0}$ is vacuum permeability and *ℏ* is the Planck constant, $\Delta N=\frac{1}{S_{R}}\frac{\hbar \omega }{k_{B}T}N$ is the molecular population difference between working levels, $S_{R}$ is the rotational partition function for all rotational levels (see Equation (9)), ω is the frequency splitting between rotational states, *r* and $L=\mu_{0}n^{2}A_{0}/l_{c}$ are the resistance and the inductance of the coil, $\Gamma =10^{7}$ $s^{-1}$ is the collisional broadening rate $\Gamma =\sigma v_{T}N_{Ar}$ and Δf is the bandwidth of detection.

The permanent electric dipole moments of NH_3_ (1.47 D) and H_2_O (1.85 D) are comparable, and the corresponding estimated detection sensitivities are therefore of the same order of magnitude. Under the Johnson–Nyquist-noise-limited assumption, the estimated number-density sensitivity for NH_3_ and H_2_O is of the order of $10^{10}\;{\mathrm{cm}}^{-3}/\sqrt{\mathrm{Hz}}$.

For the coil parameters n=10, $A_{0}=1\;{\mathrm{cm}}^{2}$, and $l_{c}=1\;\mathrm{cm}$, and for a Stark splitting corresponding to the resonant frequency $\omega =2\pi \times 10^{8}\;s^{-1}$, the estimated sensitivities are

$$N_{\min}^{{\mathrm{NH}}_{3}}=1\times 10^{10}\;{\mathrm{cm}}^{-3}/\sqrt{\mathrm{Hz}}$$

and

$$N_{\min}^{H_{2}O}=3\times 10^{10}\;{\mathrm{cm}}^{-3}/\sqrt{\mathrm{Hz}}.$$

The minimum detectable concentration estimated above represents a Johnson–Nyquist-noise-limited theoretical benchmark rather than a guaranteed experimental detection limit. In a practical implementation, additional noise sources include dielectric losses in the gas cell and surrounding materials, amplifier voltage and current noise, low-frequency 1/f noise, fluctuations of the high-voltage DC source, electromagnetic interference, and mechanical or thermal drift. Dielectric losses characterized by tanδ introduce an effective dissipative conductance $G_{\mathrm{loss}}≃\omega C\tan\delta$ and an associated thermal-noise contribution. Furthermore, fluctuations of the applied DC voltage may translate into fluctuations of the Stark-shifted rotational frequencies and may couple into the AC detection circuit. Narrow-band differential or lock-in detection at the AC modulation frequency, together with filtering and stabilization of the high-voltage source, can substantially suppress low-frequency and technical noise. Consequently, the experimentally achievable minimum detectable concentration is expected to be higher than the Johnson–Nyquist-limited value of Equation (16), with its precise value depending on the detailed circuit implementation and the measured noise spectrum.

## 3. Discussion and Conclusions

**Figure 5:**
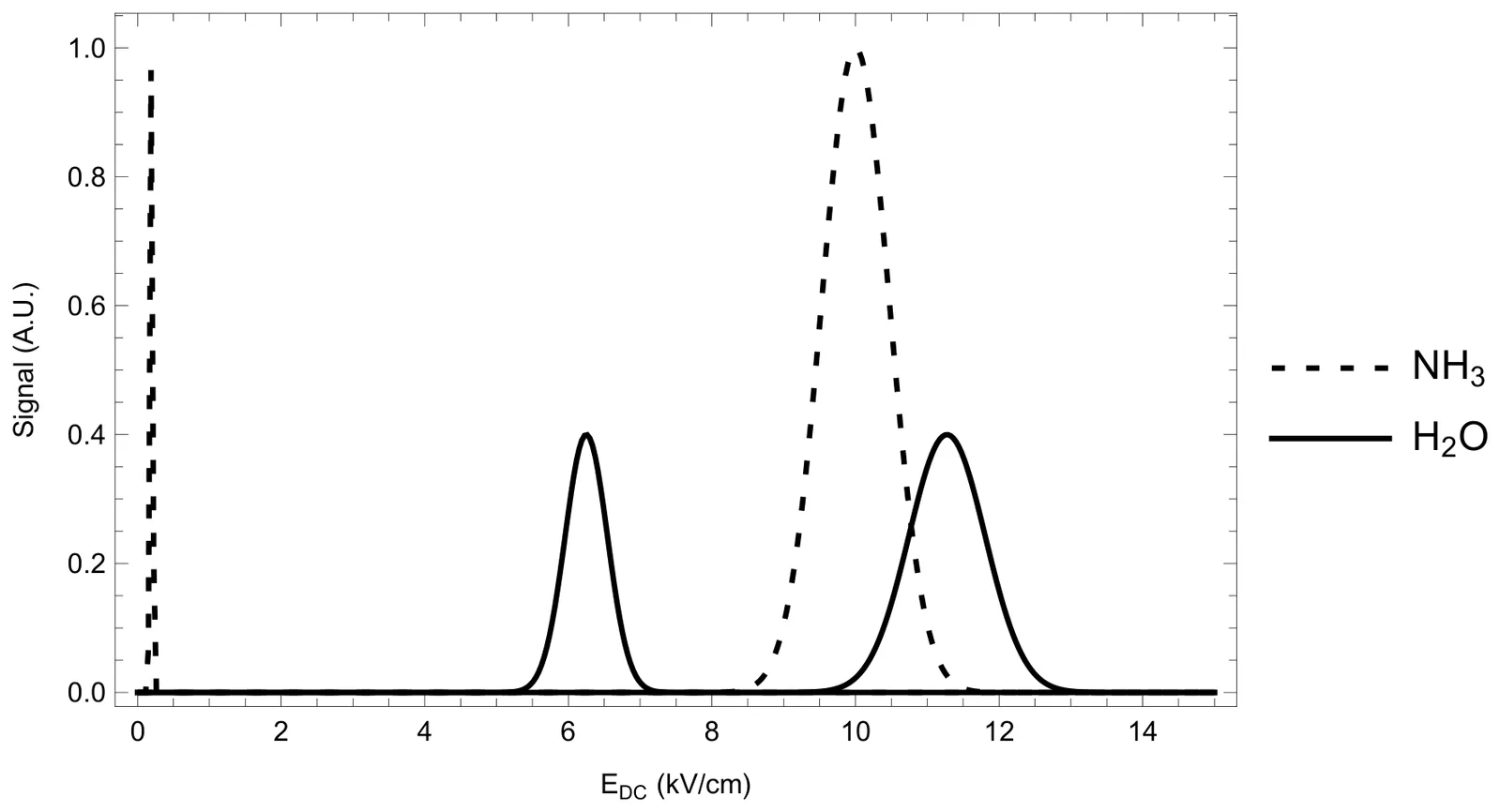
Calculated signals for NH_3_ and H_2_O, in arbitrary units, as functions of the applied DC electric field $E_{\mathrm{DC}}$. The resonance positions depend on the field-dependent molecular energy levels, while the signal amplitudes are proportional to the corresponding gas densities within the linear-response regime.

We propose a gas-sensing mechanism based on the interaction of molecular rotational states with an externally applied, quasistatically swept DC electric field. For molecules possessing a permanent electric dipole moment, the applied field produces characteristic Stark shifts and mixing of the rotational states. At a given value of the DC electric field, the resonance condition is determined by the molecular dipole moment and rotational energy structure. Consequently, when the DC field is swept while the AC excitation frequency is kept fixed, different molecular species can satisfy the resonance condition at different electric-field strengths. A gas mixture therefore produces a set of field-dependent resonance features, with each molecular species contributing its characteristic signature (see Figure 5).

This approach makes it possible, in principle, to detect multiple gases using a single excitation frequency rather than scanning over a broad frequency range. Since the positions of the resonances are determined by molecule-specific parameters, including the rotational constants, molecular symmetry, and permanent electric dipole moment, different species can be distinguished through their electric-field-dependent spectral fingerprints. The concentration of an individual species can then be inferred from the amplitude or integrated response of its associated resonance, provided that the relevant transition strengths, line broadening, and instrumental response are properly taken into account. Thus, the method offers the possibility of both qualitative identification and quantitative analysis of multicomponent gas mixtures.

Preliminary calculations for the polar molecules H_2_O and NH_3_ illustrate the feasibility of the proposed method. The widths and shapes of the resonances shown in Figure 5 are determined by the coherence relaxation rate Γ together with the field dependence of the Stark splitting summarized in Table 2. The same principle can be extended to other polar molecules, including asymmetric-top molecules, larger polyatomic species, and molecular clusters possessing sufficiently large permanent electric dipole moments and resolvable field-dependent rotational transitions. The combination of the molecular rotational structure and Stark response provides an additional dimension for molecular discrimination beyond the excitation frequency alone.

The present sensing mechanism is primarily applicable to polar molecules, since its selectivity is based on the interaction of a permanent molecular dipole moment with the applied electric field, as described by the Stark interaction in Equation (4). Molecules without a permanent electric dipole moment, such as CO_2_, N_2_, and CH_4_, can nevertheless interact with an external electric field through induced polarization. The corresponding interaction can be written as

(17) $$H_{\mathrm{ind}}=-\frac{1}{2}E\cdot \alpha \cdot E,$$

where α is the molecular polarizability tensor. In contrast to the interaction associated with a permanent dipole moment, the induced-polarization interaction is quadratic in the applied electric field. Consequently, substantially stronger DC electric fields may be required to produce energy-level shifts comparable to those obtained for polar molecules with permanent dipole moments. Although the general sensing concept could, in principle, be extended to nonpolar molecules through their field-induced polarization, a quantitative analysis of this regime is beyond the scope of the present work, which is focused on polar molecules and their Stark-shifted rotational states.

The balanced LC bridge shown in Figure 4 provides a means of detecting the small field-dependent electrical response of the gas cell. In an ideal balanced configuration, common background signals are strongly suppressed, and the differential response of the sensing cell can be measured with enhanced sensitivity. Maintaining a precisely balanced bridge in a practical instrument may, however, be technically challenging. Low-frequency modulation combined with phase-sensitive lock-in detection provides a practical approach for extracting the molecular signal in the presence of residual bridge imbalance and slow drift [25,26,27]. Active bridge balancing and periodic baseline calibration may provide additional long-term stability.

A practical implementation will also require stabilization of the LC bridge against thermal drift, mechanical perturbations, and electromagnetic interference. Thermal effects can be reduced by using components with low temperature coefficients, closely matched sensing and reference cells, and a common temperature-controlled enclosure. A rigid and symmetric cell construction, mechanically stable electrode spacing, and vibration isolation can minimize capacitance fluctuations caused by mechanical perturbations. Electromagnetic interference can be reduced by conductive shielding, appropriate grounding, shielded signal connections, and filtering and isolation of the high-voltage DC supply from the small-signal AC detection circuit. Thus, the differential bridge geometry can cancel perturbations affecting both arms, while narrow-band or lock-in detection at the AC excitation frequency can suppress broadband noise and slow drift. These engineering considerations will ultimately contribute to the practical detection limit and long-term stability of the sensor.

The reference arm of the bridge should reproduce the nonresonant background of the sensing cell as closely as possible. In a practical measurement, the reference cell can therefore contain the same carrier or background gas as the sensing cell, at approximately the same pressure and temperature, but without the target gas.

Surface interactions represent another important practical consideration, particularly for strongly polar or chemically reactive gases. Adsorption on electrodes or cell walls can lead to baseline drift, delayed desorption, memory effects, and hysteresis, while reactive species may also cause corrosion. Chemically inert and corrosion-resistant electrode materials are therefore preferable for the proposed capacitive geometry. In less chemically aggressive environments, stainless steel may provide a practical and cost-effective choice. Thin protective coatings may further reduce direct interactions between the gas and the electrode surfaces. However, such coatings must be selected carefully, as their dielectric losses and additional capacitance may affect the sensitivity and stability of the bridge.

Magnetic fields can also perturb the system through Zeeman splitting of the molecular rotational levels. For the closed-shell molecules considered here, including NH_3_ and H_2_O, the rotational Zeeman interaction is relatively weak because the associated rotational magnetic moments are typically on the scale of $\mu_{ij}$ (see Equations (7) and (8)). The characteristic frequency scale of the Zeeman interaction is

(18) $$\frac{\mu_{ij}B}{h}≃7.62\;B\;\mathrm{MHz}/T.$$

For the Earth’s magnetic field, B∼50μT, this corresponds to a characteristic frequency shift of approximately 380 Hz. This value is several orders of magnitude smaller than both the Stark shifts considered in the present sensing scheme and the characteristic transition linewidth of approximately 10 MHz, which is determined primarily by collisional broadening. Thus, under the conditions considered here, ambient magnetic fields are not expected to significantly affect the proposed sensing mechanism. For substantially narrower resonances or measurements performed in appreciable external magnetic fields, however, the Zeeman interaction can be incorporated explicitly into the molecular Hamiltonian, while appropriate magnetic shielding can reduce unwanted magnetic-field perturbations.

The sensitivity estimate presented in this work should likewise be interpreted as an ideal thermal-noise-limited benchmark. In addition to Johnson–Nyquist noise, a practical system will be affected by dielectric losses in the cell and surrounding materials, amplifier voltage and current noise, low-frequency 1/f noise, fluctuations of the high-voltage DC source, electromagnetic pickup, and residual thermal and mechanical drift. Consequently, the experimentally achievable minimum detectable concentration will generally be higher than the ideal value estimated from thermal noise alone. Narrow-band differential detection, stabilization of the DC source, careful selection of low-loss materials, and phase-sensitive detection can reduce these additional contributions.

In conclusion, the proposed method combines a single-frequency AC excitation with a controlled DC electric-field sweep to obtain molecule-specific rotational signatures without conventional broadband frequency scanning. The ability to distinguish different polar molecules through their characteristic Stark response provides a potentially useful approach to selective gas sensing and electric-field-controlled rotational spectroscopy. Applications may include environmental monitoring, breath biopsy, detection of toxic gases harmful to humans and animals, industrial process control, chemical detection, and precision spectroscopy of complex polar molecules and molecular clusters.

## Figures and Tables

**Table 1:** Permanent electric dipole moments and rotational constants for NH_3_ and H_2_O used in the calculations. The NH_3_ parameters are taken from Refs. [20,21], and the H_2_O rotational constants and dipole moment are taken from Refs. [22,23], respectively.

Molecule	Dipole Moment *℘* (C · m)	*℘* (Debye)	*A* (GHz)	*B* (GHz)	*C* (GHz)
NH_3_	$4.90\times 10^{-30}$	1.47	298.118	298.118	186.711
H_2_O	$6.17\times 10^{-30}$	1.85	835.840	435.352	278.139

**Table 2:** Calculated Stark splittings of the rotational transitions considered for NH_3_ and H_2_O. The electric-field units are indicated explicitly in each expression.

Molecule	${\mathrm{NH}}_{3}$	$H_{2}O$
Transition	Splitting Δν	
|100〉→|10±1〉	$0.75{\left(E_{0}\left[\mathrm{kV}/\mathrm{cm}\right]\right)}^{2}\;MHz$	$0.59{\left(E0[\mathrm{kV}/\mathrm{cm}]\right)}^{2}\;MHz$
|110〉→|11±1〉	$0.37\cdot E_{0}\left[V/\mathrm{cm}\right]\;MHz$	$1.92{\left(E0[\mathrm{kV}/\mathrm{cm}]\right)}^{2}\;MHz$

## Data Availability

All data supporting the findings of this study are presented within the published article.
